# Towards a critical posthumanist perspective on participatory design

**DOI:** 10.1136/medhum-2024-013078

**Published:** 2024-12-19

**Authors:** Tony Prescott, Julie M Robillard, Stuart Murray

**Affiliations:** 1 Computer Science, The University of Sheffield, Sheffield, UK; 2 Department of Medicine, Division of Neurology, University of British Columbia and BC Children's and Women's Hospital and Health Centre, Vancouver, British Columbia, Canada; 3 English, University of Leeds, Leeds, UK

**Keywords:** Philosophy, Digital Technology, Medical humanities, Community-Based Participatory Research

## Abstract

Participatory design places a strong emphasis on human agency, user perspectives and democratic ideals of inclusivity and empowerment, and is therefore often associated with humanist principles and values. In contrast, critical posthumanism questions key humanist assumptions about the centred and singular nature of the ‘human condition’. Instead, posthumanism points to the evolving and diverse lived experiences of people and how these are transformed by (and are transforming of) culture, environment and technology. In this commentary, we explore how participatory design could benefit from a posthumanist perspective that more explicitly acknowledges the entangled and interconnected nature of our technologised lives.

 Critical posthumanism is a theoretical, philosophical, political and cultural movement that proposes a reassessment of who we are, as human beings, and our relationship with other species, our planet and our machines ([Bibr R2][Bibr R3]; Wolfe 2009). Perhaps most significantly, it proposes overturning some of the classic precepts of traditional humanism concerning the centrality, rationality and autonomy of the human. Indeed, the notion that there is a ‘human condition’ is questioned. Rather, critical posthumanism asserts that there is a plurality of lived experience of humans, with no fixed point to determine our ontological status—we are both diverse and continuously evolving alongside our societies and technologies.

Posthumanism has many roots, including in philosophy (Herbrechter 2013; Roden 2015; Gunkel 2018), cultural studies, literary and film theory (Graham 2002; Murray 2019), feminism and critical race theory (Braidotti 2021; Lillvis 2017), and also the social, biological and cognitive sciences (see Nayar 2014). Related movements include sociotechnical theory (Abbas and Michael 2023) and the relational turn in sociology (Emirbayer 1997). Each of these frameworks emphasises the interconnectedness of humans both with each other and with our environments, drawing inspiration from the science of complex systems and embodied theories of cognition (Hayles 1999; Nayar 2014). In discarding notions of autonomy and ‘free will’, humans are seen as participating in complex networks of interdependency where causality is circular (Freeman 1999), emphasising the benefits of connection over notions of self-determination. This philosophical view further challenges ideas of human uniqueness, or anthropocentrism, highlighting our similarities to other animals, including in our capacity for lived experience (Nayar 2014). Biocentrism is also at issue; humans are seen as entangled and enmeshed with our technologies and therefore to be understood as hybrids or cyborgs (Haraway 1991; Prescott *et al* 2018).

Critical posthumanism is importantly different from transhumanism—the view that the human condition can be improved through science and technology—although both are sometimes viewed as strands of a broader posthumanist movement (Nayar 2014). A key difference is that transhumanism does not reject the humanist stance per se, rather it accepts the majority of its assumptions and sees technology as providing the opportunity to improve on the human condition (More and Vita-More 2013). In other words, it still privileges a particular notion of the human that critical posthumanism would see as exclusionary.

Having briefly outlined critical posthumanism, our interest in the remainder of this brief commentary is on implications of the posthumanist perspective for the design of assistive technology, and particularly for the idea of participatory design—the view that assistive technologies should be designed *with rather than for* the people they are intended to help (Sanders 2002). Participatory design is at least half-a-century old and has its roots in both the democratisation of industrial production and in product design (Sanders and Stappers 2008). In the field of assistive technology, it has been described as an interactive process in which designers and engineers engage with potential users of the technology, first to understand the needs to be addressed, and thereafter, through an iterative process, to develop and evaluate potential prototype solutions (Paulovich 2015).

Participatory design is already aligned with key ideas of critical posthumanism, particularly with regard to the embeddedness of humans in communities of connection. However, posthumanism has implications for many other central themes in participatory design, including those relating to aspiration, agency, self, dignity and the centrality of the human.

For example, a key emphasis of critical posthumanism is the rethinking of the notion of disability as explored through the discipline of disability studies (Murray 2019). Rather than viewing the body through a biomedical lens, and impairment as a deviation from ‘normal’, posthumanism sees disability as a constructed category that risks denying the right to be a differently embodied but equal citizen and stresses the generative power of disabled difference. This leads to questioning of the humanist view, sometimes implicit in participatory design approaches, that individuals with disability aspire to be closer to be the norm.

A second key theme in participatory design is an emphasis on human dignity, often described in the assistive technology literature as being promoted through independence. As already noted, posthumanism upholds a more distributed view of agency, thereby challenging traditional notions of choice and responsibility. In this context, dignity is viewed as relational and dynamic, and not primarily dependent on autonomy.

Finally, a posthuman approach broadens the outlook of participatory design from a Western-focused cultural, anthropocentric and biocentric one to one that acknowledges wider perspectives, including those that bring into consideration Global South and Indigenous societies, non-human entities including animals and ecosystems, and future technological systems including, potentially, those involving robots and artificially intelligent (AI) agents.

Posthumanist views are already impacting on participatory design through a variety of methodologies, such as those inspired by sociotechnical and relational viewpoints (see other contributions in this issue). Ongoing posthumanist research is also exploring how our aspirations are shaped by our interactions with current technologies, philosophical configurations of selfhood and by imaginaries about our future, such as those emerging from science fiction.

As one example, research conducted within the Wellcome Trust ‘Imagining Technologies for Disability Futures’ project explores the consequences of a posthumanist view for the development of companion AIs and robots as assistive technologies (see also Gunkel (2018) and Stimson (2024) in this issue). Some humanist perspectives (eg, Turkle 2017) assert that to present a synthetic device as able to provide companionship is simply unethical as artefacts, however complex, are intrinsically incapable of being ‘truly social’. In contrast, posthumanism implies a more consequentialist view, consistent with participatory design methods, that seeks to understand through user engagement the potential meaning, value and significance of synthetic companions. What is unethical then depends on how the use of such technologies impacts on those involved and their wider societal and relationship settings (Prescott and Robillard 2021). Key design issues include the capacity of the technology to adapt to different individuals and their contexts, and to clearly signal its own capabilities and differences (Prescott and Robillard 2022). The use of human-like characteristics (anthropomorphism) is not seen as intrinsically deceptive, but is understood in a more nuanced way—anthropomorphic qualities are seen as positive when they promote valued interaction but as negative when used to deliberately mislead or misdirect (Danaher 2019; Damiano and Dumouchel 2018; Prescott and Robillard 2022).

Looking forward, we can expect that existing boundaries between self and other, and human and machine, may further evolve, or even partially dissolve, through technologies such as virtual reality, cognitive prostheses and brain–machine interfaces. While rejecting both technosolutionism and the traditional duality of replacement versus augmentation, the posthuman approach recognises the potential for momentous change, due to emerging technologies such as these, whose consequences and impacts we should explore and understand through participatory methods.
